# Feasibility and Safety of an Eight-Week Exercise Program with the Additional Peripheral Magnetic Stimulation of the Abdominal Muscles

**DOI:** 10.3390/healthcare12141434

**Published:** 2024-07-18

**Authors:** Denisa Manojlović Ivezić, Jure Žitnik, Nejc Šarabon

**Affiliations:** 1 Faculty of Health Sciences, University of Primorska, Polje 42, SI-6310 Izola, Slovenia; denisa.manojlovic@fvz.upr.si (D.M.I.); jure.zitnik@fvz.upr.si (J.Ž.); 2 Human Health Department, InnoRenew CoE, Livade 6, SI-6310 Izola, Slovenia; 3 Laboratory for Motor Control and Motor Behavior, S2P, Science to Practice, Ltd., Tehnološki Park 19, SI-1000 Ljubljana, Slovenia; 4 Ludwig Boltzmann Institute for Rehabilitation Research, Neugebäudeplatz 1, 3100 St. Pölten, Austria

**Keywords:** feasibility, safety, magnetic stimulation, abdominal wall

## Abstract

Peripheral magnetic stimulation has recently been introduced as a non-invasive but effective physical agent to improve muscle strength and everyday function. The aim of this study was to evaluate the feasibility and safety of an exercise program focusing on the abdominal muscles in combination with the peripheral magnetic stimulation of the abdominal muscles. Male and female overweight and obese adults (n = 19) participated in an eight-week exercise program with the additional peripheral magnetic stimulation of the abdominal muscles. Outcome measures included changes in abdominal subcutaneous fat thickness, trunk muscle strength, body composition, and self-reported body satisfaction. Subcutaneous fat thickness was significantly reduced after the intervention (*p* < 0.01–*p* < 0.001). Trunk flexion and left side flexion strength increased significantly after the intervention, although no significant changes were observed for trunk extension (*p* = 0.07) and right side flexion strength (*p* = 0.13). The body satisfaction self-assessment score significantly increased (*p* < 0.01), while body mass, body mass index, and fat mass significantly decreased after the intervention (*p* < 0.05). Our findings suggest that an exercise program with the additional peripheral magnetic stimulation is feasible and safe for overweight and obese participants. These results support the use of peripheral magnetic stimulation as a safe adjunct to the voluntary abdominal muscle contraction. Future studies are needed to evaluate the efficacy of the additional peripheral magnetic stimulation of the abdominal muscles compared to the voluntary contraction of the abdominal muscles alone.

## 1. Introduction

Excess body mass is a global public health problem, as more than half of the adult population is overweight or obese [1,2]. In addition to a significant economic burden on the health care system [3], overweight and obesity are associated with type II diabetes, cardiovascular and musculoskeletal disorders, cancer, asthma, and premature mortality [4]. In addition, excess body mass places stress on the musculoskeletal system, even during everyday tasks, and increases the risk for various injuries. Consequently, pain caused by various musculoskeletal disorders can affect muscle strength, reduce the physical activity level, and further increase the risk of excess body mass [5]. Thus, novel and innovative approaches are emerging with the aim to reduce the burden that excessive body mass has on the individual and the public health system.

Exercise interventions have been widely accepted as one of the most effective ways for the prevention of obesity and reduction in excess body mass. Moreover, various passive agents are increasingly investigated to enhance the effectiveness of exercise programs. Although electrical muscle stimulation systems have been traditionally used as a means to augment voluntary muscle contraction, its applicability and adverse effects limit its use in clinical practice [6]. Furthermore, the feasibility and safety of electrical muscle stimulation may be limited by the inevitable stimulation of the nociceptors in the skin and the requirement of a direct contact between the skin and the electrode. For these reasons, various novel physical agents have been proposed in order to upgrade everyday clinical practice combining evoked muscle contractions with active movement. Peripheral magnetic stimulation (PMS) has recently been proposed as an effective non-invasive physical agent to improve everyday function and muscle strength in patients with neurological, urogenital, and musculoskeletal disorders [6,7,8,9]. Unlike electrical muscle stimulation, PMS generates electrical current in the tissue without overstimulating nociceptors in the skin [6] and may reach tissues as deep as seven centimeters [10]. It is assumed that due to its aforementioned characteristics, PMS may present a valuable tool in enhancing muscle contractions. Since its introduction into clinical and research practice, PMS has been shown to be a safe and efficient treatment for a wide range of patients [7] and has been most frequently used in the treatment of urogenital disorders [11,12]. However, there is evidence suggesting that PMS improves the strength of the suprahyoid [13] and quadriceps muscles [14,15]. The improvement in muscle strength observed following a PMS intervention has been attributed to various factors, including greater muscle activation, neural adaptation, and an increase in the thickness of the targeted muscle group [16,17]. However, these findings are not universal throughout the literature. Yang et al. [14] report an increase in quadriceps muscle strength with no significant difference in muscle thickness during a five-week quadriceps strengthening intervention, whereas Abulhasan et al. [15] concluded that PMS could help with performing additional muscle contractions, without a significant effect on muscle strength and thickness. Despite the focus on muscle strength in previous studies using PMS, the effect of PMS on subcutaneous fat around the stimulated muscles has received little attention. It has been suggested that PMS may lead to increased effectiveness of exercise programs due to a reduction in excessive subcutaneous fat. Recent studies have demonstrated a 23% decrease in subcutaneous fat thickness [10] and a 3.26 cm decrease in waist circumference [18] following a 12-week PMS intervention. Accordingly, in a study by Fabi et al. [19], 68% of participants were found to have an increase in body satisfaction on the Body Satisfaction Questionnaire at the 12-week follow-up, indicating a possible influence of the PMS intervention on the quality of life. However, the feasibility and safety of an eight-week exercise program with the added PMS of the abdominal muscles have yet to be examined.

Therefore, the aim of this study was to evaluate the feasibility and safety of an eight-week exercise program focusing on the abdominal muscles in combination with PMS of the abdominal muscles. With regards to the aforementioned limitations of electrical muscle stimulation, we hypothesized that the addition of PMS to the voluntary contraction of the abdominal wall muscles would be tolerable and safe for all participants. Our evidence on the safety and tolerability of PMS could help with closing the knowledge gap regarding the adjunct of passive physical agents to voluntary muscle contractions. With consideration to previous studies [10], we further hypothesized that PMS would effectively reduce subcutaneous fat thickness, improve muscle strength, and increase body satisfaction with the aim to direct future high-quality studies.

## 2. Materials and Methods

This was a prospective, open-label feasibility study. The study protocol was approved by the Slovenian Medical Ethics Committee (0120-369/2021/7, 28 October 2021) and was conducted according to the Declaration of Helsinki. This study was registered on clinicaltrials.gov (NCT05524636).

### 2.1. Participants

Participants were invited to participate in this study via invitations shared through the local radio station and through social media that included basic information about this study. Predominantly sedentary adults, between the ages of 18 and 50, were eligible for participation in this study. Participants were eligible for this study if they also met the following inclusion criteria: (a) a body mass index (BMI) greater than 25.0 kg/m^2^, (b) absence of change in body mass during the month prior to the inclusion in this study, and (c) waist circumference >80 cm for women and >90 cm for men [10,20]. Participants were excluded from this study if they (a) had an ongoing musculoskeletal or neuromuscular injury or condition, (b) had received any invasive fat reduction treatment procedure (e.g., mesotherapy or liposuction), (c) had taken dietary weight loss supplements one month prior to study enrollment, (d) had a metal implant, or (e) had an active electrical implant (e.g., pacemaker or hearing aid) [19]. All eligible participants gave their informed and written consent before enrollment in this study.

### 2.2. Intervention

Participants took part in a supervised, eight-week exercise program focusing on the abdominal muscles and augmented by the additional PMS of the abdominal muscles. Each exercise session lasted 45 min and was performed 3×/week with at least one day between sessions. The program included a specific warm-up, followed by widely spread exercises for increasing abdominal muscle strength and endurance. The session was concluded with a general cool-down and relaxation (Table 1). Semi-static exercises were chosen to ensure safe and effective PMS of the abdominal muscles. The quantity and intensity of the exercises were progressively increased during the intervention period.

A novel commercial device was used to deliver PMS (Star Former, Fotona Ltd., Ljubljana, Slovenia). The strength of the magnetic field generated by the Star Former device reaches up to 3 Tesla and is delivered by biphasic pulses of a 300 ms duration. The frequency of the magnetic pulse varied between 20 Hz and 30 Hz and occurred simultaneously with the voluntary contraction of the abdominal muscles [9]. The initial intensity of the PMS was set at 5% of the maximal output of the device and was progressively increased to the maximum tolerance of each participant [15]. To ensure the constant stimulation of the abdominal muscles during voluntary contractions, the applicator was attached to the treatment area with a custom-made fixation strap. The circular coil of the applicator was placed on the umbilicus to ensure the optimal stimulation of the rectus abdominis muscle and the transversus abdominis muscles [10], and was moved sideways during exercises focusing on the external and internal oblique muscles to ensure the optimal stimulation of the activated muscles. All jewelry was removed from the treatment area prior to the treatment, while the impulses were delivered through clothing. The safety of the intervention protocol was carefully monitored during each exercise session, and any device-related adverse effects were noted. Participants were asked to report any discomfort or pain during or after the exercise session.

### 2.3. Assessments

All assessments were supervised and/or performed by researchers with experience in the field of physiotherapy and sports. Participants were assessed at baseline (Pre) and eight weeks after baseline (Post) by the same research group. The time of each participant’s baseline assessment was noted to schedule subsequent assessment at approximately the same time (±2 h) [15]. This was conducted to reduce the effects of individual circadian rhythms as much as possible. 

**Figure 1:**
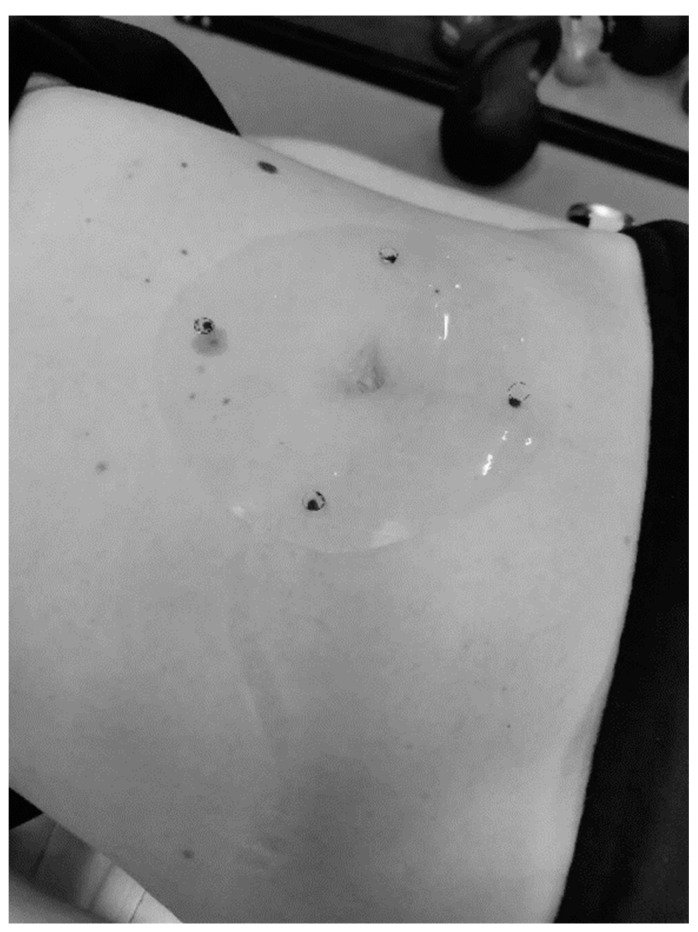
Template of ultrasound measurement points.

The primary outcome was the change in abdominal subcutaneous fat thickness measured by diagnostic ultrasound (US). The US measurements were performed using the Resona 7 US system (Mindray Bio-Medical Electronics Co., Ltd., Shenzhen, China). Subcutaneous fat thickness measurements were performed by the same researcher at both time points. Participants were positioned supine on the examination table with their legs extended and arms at their sides. To ensure consistency, a template was used on which the measurement points were marked (Figure 1). The measurement points were positioned 5 cm superiorly, inferiorly, and laterally on both sides of the umbilicus [10]. The US assessment was performed using the L11-3U linear probe with a frequency range of 3.0 MHz to 10.0 MHz. A thick layer of US gel was used to minimize the pressure of the probe on the skin surface. All US images were reviewed and analyzed by two researchers, to ensure the reliability of the measurements. Body mass, fat-free mass, fat mass, and body fat percentage were assessed using a multi-frequency bioelectrical impedance device (Tanita MC-980MA, Tanita Corp., Tokyo, Japan) at both the Pre and Post visit. Height was measured to the nearest 0.1 cm (Seca 213, Seca GmbH & Co. KG, Hamburg, Germany) following enrollment into the investigation at the Pre visit.

**Figure 2:**
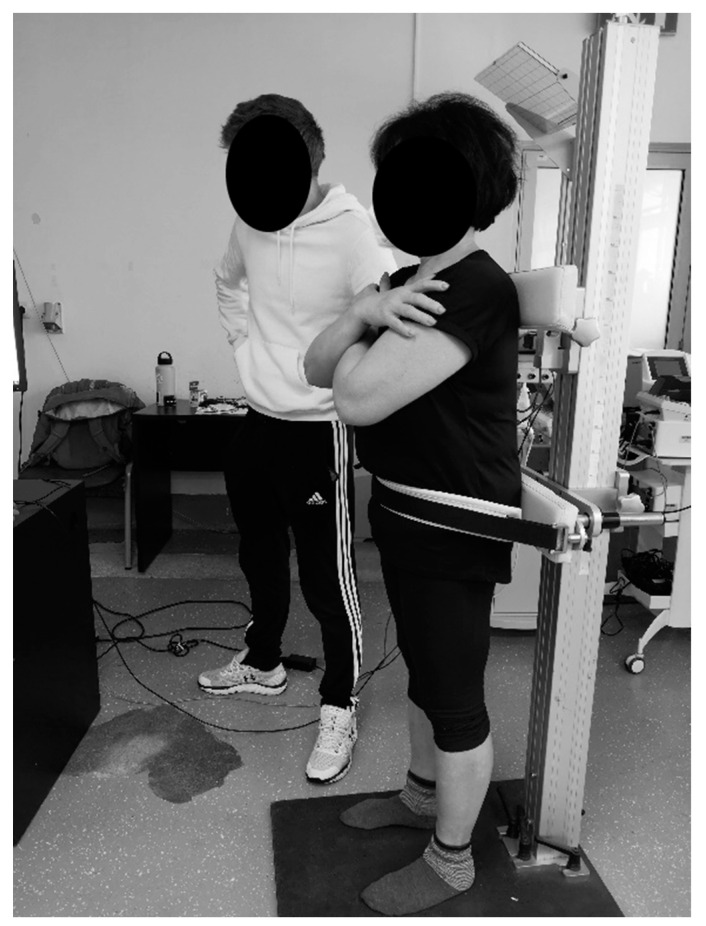
Maximal voluntary isometric contraction of trunk extension.

Secondary outcomes were trunk muscle strength and self-reported body satisfaction. For strength assessments, a custom-made dynamometer was used to perform maximal voluntary isometric contractions of the trunk flexion, extension, and both side flexions. Participants were positioned on the dynamometer platform with the sensor at shoulder height and changed their orientation depending on the muscle group being assessed. Participants performed three repetitions of each contraction while being verbally encouraged to push as hard as possible against the dynamometer’s sensor [21]. The measurement was supervised and conducted by two researchers; one monitoring the signals from the dynamometer, and the other assisting the participant during the orientation changes (Figure 2). The signals were exported, analyzed, and reported as torque normalized to body mass (Nm/kg). Participants were additionally asked to assess their subjective body satisfaction by ticking their level of agreement with the statement “I am satisfied with my body image” on a Likert scale ranging from 1 (strongly disagree) to 5 (strongly agree) [10].

### 2.4. Statistical Analyses

Data were collected in prepared Excel spreadsheets. Descriptive statistics were calculated for all outcome variables. The distribution of each of the outcome variables was verified by the Shapiro–Wilk test and visual inspection of QQ plots. The Pre–Post comparisons were performed using the Student’s paired *t*-test and, if the assumptions for the paired *t*-test were not met, the Wilcoxon signed-rank test. Additionally, 95% confidence intervals (CIs) and Cohen’s d effect sizes were calculated for the mean differences. Cohen’s d effect sizes were interpreted as follows: small (0.20–0.50), moderate (0.50–0.80), and large (>0.80) [22]. For the Wilcoxon signed-rank test, the r effect size was calculated and interpreted as follows: small (0.10–0.30), moderate (0.30–0.50), and large (>0.80) [23]. An a priori power analysis using G*Power 3.1.9.7 [24] indicated a minimum sample size of 15 to achieve 80% power for detecting a large effect with Student’s paired *t*-test and the Wilcoxon signed-rank test (α = 0.05). The statistical significance for all analyses was set at *p* < 0.05. All analyses were performed with the program R Studio (version 2022.07.1, R Core Team, 2022).

## 3. Results

A total of 22 participants volunteered to participate in this study. No participant reported any pain or discomfort due to PMS of the abdominal muscles during or after the exercise sessions. An eight-week exercise program focusing on the abdominal muscles with the added PMS seems to be feasible and safe for all participants. By the end of the eight-week period, three participants have withdrawn from this study for personal reasons. The baseline characteristics of participants are presented in Table 2.

### 3.1. Body Composition

Body mass, BMI, and fat mass significantly decreased after the intervention, while no significant changes were observed for fat-free mass (*p* = 0.15) and body fat percentage (*p* = 0.07) (Table 3). Moderate effect sizes were found for changes in body mass and BMI, while the effect size for changes in fat mass was small.

### 3.2. Subcutaneous Fat Thickness

**Figure 3:**
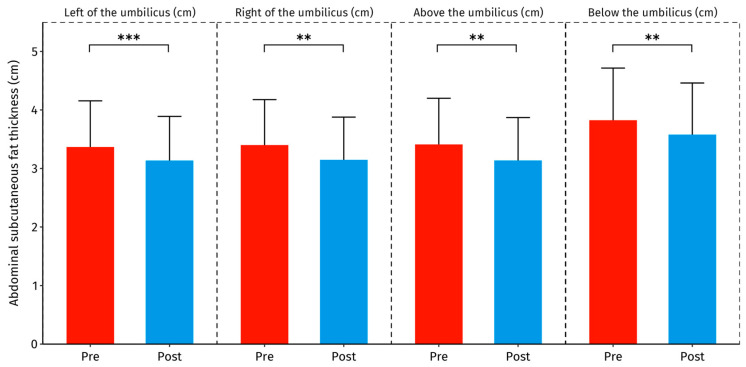
Changes in subcutaneous fat thickness around the umbilicus before (Pre) and after the intervention (Post). Columns represent the mean ± SD with error bars denoting 95% CI. **—*p* < 0.01, ***—*p* < 0.001.

Subcutaneous fat thickness was significantly reduced after the intervention at all four abdominal measurement sites (Table 3 and Figure 3). The Pre–Post mean difference ranged from −0.23 cm to the left of the umbilicus to −0.27 cm above the umbilicus (*p* < 0.01–*p* < 0.001). The effect size was large for changes in subcutaneous fat thickness below the umbilicus, and lateral on both sides of the umbilicus, while there was a moderate effect size shown for changes in subcutaneous fat thickness above the umbilicus.

### 3.3. Trunk Muscle Strength

**Figure 4:**
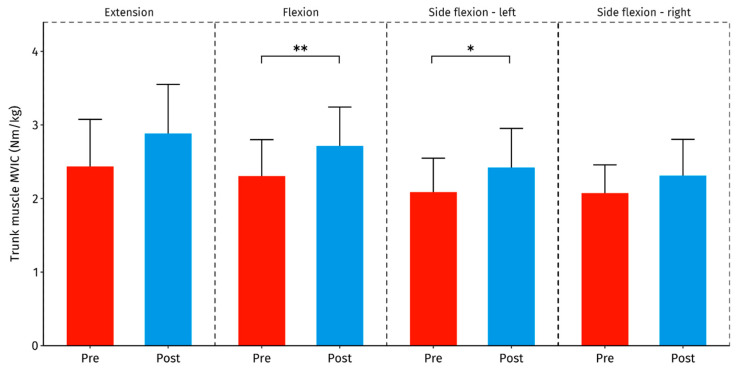
Changes in trunk muscle strength before (Pre) and after the intervention (Post). Columns represent the mean ± SD with error bars denoting 95% CI. *—*p* < 0.05, **—*p* < 0.01.

Trunk flexion and left side flexion strength increased significantly after the intervention (corresponding to a moderate effect size in both cases), while no statistically significant changes were observed for trunk extension (*p* = 0.07) and right side flexion strength (*p* = 0.13) (Table 4 and Figure 4).

### 3.4. Body Satisfaction Score

The body satisfaction self-assessment score was significantly increased after the intervention (z = 2.977, *p* < 0.01; r = 0.68), with a mean increase of 1 point (95% CI: 1.0–1.5) on the Likert scale, yielding a large effect size.

## 4. Discussion

The main purpose of our study was to evaluate the feasibility and safety of an eight-week exercise program focusing on the abdominal muscles with the additional PMS of the abdominal muscles. We also wanted to investigate the effectiveness of the exercise program with the addition of PMS in terms of subcutaneous fat thickness, body composition, trunk muscle strength, and body satisfaction. To the best of our knowledge, this was the first study to examine the feasibility and safety of an eight-week exercise program with added PMS focused on the abdominal muscles.

The results of our study suggest that an eight-week exercise program with additional PMS of the abdominal muscles is feasible and safe for overweight adults and is associated with improvements in body composition, subcutaneous fat thickness, muscle strength, and body satisfaction. These findings are consistent with previous studies confirming the safety of PMS for abdominal contouring [18,25]. However, it is worth noting that the PMS intervention in the aforementioned studies was delivered in four sessions over two weeks. Although these results provided valuable information on the short-term effects of PMS on abdominal contouring, the safety and feasibility of a longer intervention including PMS and active exercise remained unknown. The results of our study add to the knowledge of the safety of PMS through an eight-week intervention combining PMS with voluntary contractions of the abdominal muscles, showing safety and feasibility over a longer intervention period. Altogether, three participants withdrew from this study for personal reasons. The PMS was successfully applied over the course of the eight-week program, and no side effects were noted by participants or investigators. None of the included participants communicated any pain or discomfort due to PMS of the abdominal muscles during or after the exercise sessions. It therefore appears that the addition of PMS of the abdominal muscles is feasible and safe to be implemented in larger randomized controlled trials.

Our results demonstrated some improvements in body composition during the intervention period. A recent study conducted by Fabi et al. [19] indicated that PMS may contribute to the contouring of abdominal area by stimulating the underlying muscles and reducing the waist circumference by up to 1.1 cm. Accordingly, our results showed a statistically significant reduction in body mass, BMI, and fat mass during the intervention period, while no significant changes were observed in fat-free mass and body fat percentage. It should be noted that our study primarily investigated the feasibility and safety of an eight-week exercise program with the additional PMS of the abdominal muscles, and no other lifestyle modification aimed specifically at improving body composition was employed in this study. Nevertheless, these results provide a valuable starting point for future research examining the effectiveness of PMS on body composition. In addition, participants in our study rated their satisfaction with their bodies as significantly improved after the intervention. These results are in concordance with previous evidence, reporting that 91% of participants were satisfied after an intervention employing four PMS treatments [10].

Few studies examined changes in subcutaneous fat thickness after PMS intervention using US. Katz et al. [10] reported a decrease in mean subcutaneous fat thickness of −0.34 cm to −0.63 cm at the same measurement points after four PMS treatments, whereas our results demonstrated a mean reduction in subcutaneous fat thickness in the abdominal wall area up to −0.27 cm. Although a reduction in subcutaneous fat thickness was observed in our study, it was notably smaller (averaged over four measurement points) than the average reduction over four measurement points reported by Katz et al. [10] (0.25 vs. 0.45 cm), with a further discrepancy when the reduction in thickness is expressed in relative values (8% vs. 19%). This is somewhat surprising, considering the shorter study duration with fewer PMS sessions, and the absence of an exercise intervention in the aforementioned study. Although body composition was not assessed by Katz et al., it is likely that intervention was accompanied by significant reductions in total body fat in addition to abdominal reductions in fat thickness. However, it is worth noting that neither our study design nor the study design used by Katz et al. allows one to discern the effect of PMS independently of the other factors, such as negative energy balance resulting in weight loss and decrease in whole-body subcutaneous fat thickness. It should also be noted that there is a lack of evidence supporting localized direct effects of PMS on fat cells as evidenced by the absence of fat cell injury or local inflammation in response to a single 30 min treatment [26].

The observed increases in trunk muscle strength were expected and probably occurred mainly due to the exercise program employed in the intervention. However, it should be noted that the role of PMS in observed increased trunk muscle strength cannot be discerned from the effect of the exercise program employed in our study. Our results provide valuable information about the feasibility of the exercise program with and without PMS and changes in strength that occurred during an eight-week exercise program with the additional PMS of the abdominal muscles, but clear conclusions about the true effectiveness of PMS on muscle strength remain to be drawn by larger randomized controlled trials. 

Several limitations should be noted when interpreting the results of this study. First, our study included non-active and overweight and obese participants. These criteria were established to assess the feasibility of our protocol in this population, as the beneficial health effects would be particularly relevant to these groups if the protocol was proven as feasible. However, it is not reasonable to infer the safety of PMS of the abdominal muscles in a general and more physically active population with a thinner layer of subcutaneous fat in the abdominal area, solely from the absence of reported side effects in our study. Further studies should therefore carefully investigate the feasibility and safety of different exercise programs with the addition of PMS of the abdominal muscles in normal weight participants. Second, a major limitation of our study is the lack of a control group. To investigate the efficacy of the additional PMS of the abdominal muscles, a control group performing the exercise program without the additional PMS should be included. Third, no controlled dietary intervention was included in our study. Controlling for energy intake would allow us to exclude the possible effect of a negative energy balance on the changes in body composition and subcutaneous fat thickness. Therefore, the reported effects on body composition and subcutaneous fat thickness in this study should not be interpreted as a direct causal effect of the additional PMS. However, the main objective of the current study was to evaluate the feasibility and safety of the exercise program coupled with abdominal PMS. Control of diet and other lifestyle factors presents an opportunity for future controlled studies.

## 5. Conclusions

Our study indicates that an eight-week exercise program focusing on abdominal muscles with the additional PMS of the abdominal muscles is feasible and safe for overweight and obese participants. Changes in body mass, BMI, fat mass, and subcutaneous fat thickness as well as increases in trunk muscle strength and body satisfaction were observed. However, these conclusions should be considered with caution due to various limitations of our study. Future studies should evaluate the effectiveness of the additional PMS of the abdominal muscles compared to the voluntary contraction of the abdominal muscles alone.

## Figures and Tables

**Table 1:** Exercise program focusing on the abdominal muscles.

	W1	W2	W3	W4	W5	W6	W7	W8
	The Warm-Up Cycle Is Repeated 2×							
Hip circles	30 s	30 s						
Low skip	30 s	30 s	30 s	30 s	30 s	30 s	30 s	30 s
High skip	30 s		30 s		30 s		30 s	
Jumping jacks	30 s	30 s		30 s		30 s		30 s
Standing side crunches	30 s	30 s	30 s		30 s	30 s	30 s	30 s
Standing crunches		30 s	30 s	30 s		30 s	30 s	30 s
Pop-squat		30 s	30 s	30 s		30 s	30 s	
Lunges			30 s	30 s	30 s		30 s	
Squatting side walk				30 s	30 s	30 s		
	W1	W2	W3	W4	W5	W6	W7	W8
Short crunches *	3 × 8	3 × 10						
Short crunches with rotation *	3 × 8	3 × 10						
High plank *	3 × 15 s	3 × 20 s						
Side crunches with knees flexed I *	3 × 15 s	3 × 20 s	3 × 25 s	3 × 30 s				
Side crunches with knees flexed II *	3 × 15 s	3 × 20 s	3 × 25 s	3 × 30 s				
Pilates ball sitting with leg rise I *	3 × 15 s	3 × 20 s	2 × 30 s	2 × 45 s				
Pilates ball sitting with leg rise II *	3 × 15 s	3 × 20 s	2 × 30 s	2 × 45 s				
Pilates ball sitting with pelvic tilt *	3 × 15 s	3 × 20 s	2 × 30 s	2 × 45 s				
Plank *			3 × 15 s	3 × 20 s	3 × 25 s	3 × 30 s		
Short crunches on Pilates ball *			3 × 8	3 × 10	3 × 12	3 × 15		
Plank with alternate leg rise *					3 × 8	3 × 10	3 × 12	3 × 15
Trunk rotations on Pilates ball *					3 × 8	3 × 10	3 × 12	3 × 15
Plank on Pilates ball *					3 × 15 s	3 × 20 s	3 × 25 s	3 × 30 s
Side plank with knees flexed I *					3 × 15 s	3 × 20 s	3 × 25 s	3 × 30 s
Side plank with knees flexed I *					3 × 15 s	3 × 20 s	3 × 25 s	3 × 30 s
Mountain climbers *							3 × 15 s	3 × 20 s
Trunk rotation with leg rise *							3 × 15 s	3 × 20 s
Abdominal muscle stretch on forearms	30 s	30 s	30 s	30 s	30 s	30 s	30 s	30 s
Abdominal muscle stretch on Pilates ball	30 s	30 s	30 s	30 s	30 s	30 s	30 s	30 s
Lying side body stretch I	30 s	30 s	30 s	30 s	30 s	30 s	30 s	30 s
Lying side body stretch II	30 s	30 s	30 s	30 s	30 s	30 s	30 s	30 s

W: week, *: exercise with additional peripheral magnetic stimulation.

**Table 2:** Baseline characteristics of participants.

Outcome Measure	3 M, 16 F (n = 19)
Age (years)	44.4 ± 4.9
Body height (cm)	169.3 ± 8.7
Body mass (kg)	90.8 ± 21.8
Body mass index (kg/m^2^)	31.8 ± 8.1
Body fat percentage (%)	35.6 ± 8.6
Fat mass (kg)	33.6 ± 15.0
Fat-free mass (kg)	57.2 ± 10.1

Data presented as mean ± SD; M—male; F—Female.

**Table 3:** Changes in body composition and abdominal subcutaneous fat thickness before (Pre) and after the intervention (Post).

	Pre	Post	Pre–Post Mean Difference (95% CI)	Cohen’s d	Magnitude
Body composition					
Body mass (kg)	90.8 ± 21.8	88.9 ± 20.8	−1.86 (−3.29–−0.42) *	0.62	moderate
Body mass index (kg/m^2^)	31.8 ± 8.1	31.2 ± 8.0	−0.59 (−1.05–−0.12) *	0.61	moderate
Fat-free mass (kg)	57.2 ± 10.1	56.9 ± 10.3	−0.36 (−0.87–0.14)	0.35	small
Fat mass (kg)	33.6 ± 15.0	32.1 ± 14.1	−1.49 (−2.91–−0.08) *	0.51	moderate
Body fat percentage (%)	35.6 ± 8.6	34.8 ± 8.3	−0.77 (−1.60–0.06)	0.44	small
Abdominal subcutaneous fat thickness					
Left of the umbilicus (cm)	3.37 ± 1.64	3.14 ± 1.57	−0.23 (−0.35–−0.11) ***	0.91	large
Right of the umbilicus (cm)	3.40 ± 1.61	3.15 ± 1.52	−0.25 (−0.39–−0.11) **	0.87	large
Above the umbilicus (cm)	3.41 ± 1.64	3.14 ± 1.52	−0.27 (−0.46–−0.09) **	0.73	moderate
Below the umbilicus (cm)	3.83 ± 1.85	3.58 ± 1.83	−0.25 (−0.39–−0.10) **	0.80	large

Data presented as mean ± SD; CI—confidence interval; *—*p* < 0.05, **—*p* < 0.01, ***—*p* < 0.001.

**Table 4:** Changes in trunk muscle strength before (Pre) and after the intervention (Post).

	Pre	Post	Pre–Post Mean Difference (95% CI)	Cohen’s d	Magnitude
**Trunk muscle MVIC**					
Extension (Nm/kg)	2.44 ± 1.33	2.88 ± 1.39	0.44 (−0.04–0.93)	0.44	small
Flexion (Nm/kg)	2.31 ± 1.03	2.71 ± 1.10	0.41 (0.11–0.71) **	0.67	moderate
Side flexion—left (Nm/kg)	2.09 ± 0.96	2.42 ± 1.1	0.33 (0.06–0.61) *	0.59	moderate
Side flexion—right (Nm/kg)	2.07 ± 0.80	2.31 ± 1.03	0.24 (−0.08–0.55)	0.36	small

Data presented as mean ± SD; CI—confidence interval; *—*p* < 0.05; **—*p* < 0.01.

## Data Availability

The original contributions presented in the study are included in the article; further inquiries can be directed to the corresponding author.
